# Maslinic Acid Ameliorates Inflammation via the Downregulation of NF-κB and STAT-1

**DOI:** 10.3390/antiox9020106

**Published:** 2020-01-25

**Authors:** Wonhwa Lee, Jaehong Kim, Eui Kyun Park, Jong-Sup Bae

**Affiliations:** 1 College of Pharmacy, CMRI, Research Institute of Pharmaceutical Sciences, BK21 Plus KNU Multi-Omics based Creative Drug Research Team, Kyungpook National University, Daegu 41566, Korea; bywonhwalee@gmail.com; 2 Aging Research Center, Korea Research Institute of Bioscience and Biotechnology (KRIBB), Daejeon 34141, Korea; 3 Department of Biochemistry, College of Medicine, Gachon University, Incheon 21999, Korea; geretics@gachon.ac.kr; 4 Department of Pathology and Regenerative Medicine, School of Dentistry, Kyungpook National University, Daegu 41940, Korea; epark@knu.ac.kr

**Keywords:** maslinic acid, endothelium, iNOS, p-STAT-1

## Abstract

Maslinic acid (MA), a natural compound of the triterpenoid group derived from olive, prevents the generation of pro-inflammatory cytokines and oxidative stress. In human umbilical vein endothelial cells (HUVECs) treated with lipopolysaccharide (LPS), we characterized the effects of MA on the regulation of heme oxygenase (HO)-1, cyclooxygenase (COX-)2, and inducible nitric oxide synthase (iNOS). MA was tested in the lung tissues of LPS-treated mice, to determine its effect on levels of iNOS expression and representative inflammatory mediators such as interleukin (IL)-1α and tumor necrosis factor (TNF)-α. We show that MA induced the expression of HO-1, reduced LPS-induced NF-κB-luciferase activity, and inhibited iNOS/NO and COX-2/PGE2, resulting in the downregulation of STAT-1 phosphorylation. Furthermore, our data show that MA induced the nuclear translocation of Nrf2, increased the binding of Nrf2 to ARE, and decreased IL-1α production in LPS-treated HUVECs. The MA-induced reduction in iNOS/NO expression was reversed by RNAi suppression of HO-1. In mice treated with LPS, MA significantly downregulated levels of iNOS in lung tissue and TNF-α in the bronchoalveolar lavage fluid. Taken together, our findings indicate that MA exerts a critical anti-inflammatory effect by modulating iNOS via the downregulation of NF-κB and p-STAT-1. Thus, we propose that MA may be an ideal substance to treat inflammatory diseases.

## 1. Introduction

In response to oxidative stress and inflammatory injury, heme oxygenase-1 (HO-1) considerably influences the progression of a variety of severe diseases, such as lung disease, systemic autoimmune disease, and cancer [1,2]. HO-1 suppresses the synthesis of inflammatory mediators such as tumor necrosis factor (TNF)-α, interleukin (IL)-1β, and IL-6 [2]. HO-1 has been shown to have a protective effect against sepsis in ‘Cecal ligation and puncture’-induced sepsis in mice models [2,3]. HO-1 has been successfully used to treat various vascular inflammatory disorders [2]. The expression of the HO-1 gene is regulated by nuclear factor erythrocyte 2-related factor 2 (Nrf2) that binds to the antioxidant response elements (AREs) on the promoters of the antioxidant enzyme genes. The expression of the antioxidant enzymes is triggered in response to oxidative and other forms of stress [4]. The Nrf2-ARE pathway is thus considered an important target in the treatment of inflammatory diseases [4]. 

Maslinic acid (MA, 2-α,3-β-dihydroxyolean-12-en-28-oic acid) is a natural compound of the triterpenoid group, derived from olive, and is known by the botanical name Olea europaea, meaning European olive. MA is also found in a variety of medicinal plants [5,6]. MA has been shown to have antimalarial [7], antiprotozoan [8], antioxidant [9], and anti-inflammatory [10] activities. However, it is not known how MA regulates HO-1 and inflammatory mediators such as NO, IL-1β, and TNF-α in endothelial cells in vitro or in mice tissue stimulated with lipopolysaccharide (LPS). In our study, we determined the effects of MA on the induction of HO-1 and the reduction of inflammatory mediators. We further explored the mechanism by which MA acts as an anti-inflammatory compound.

## 2. Materials and Methods 

### 2.1. Cell Culture and Reagents 

Human umbilical vein endothelial cells (HUVECs) were obtained from Cambrex Bio Science (Charles City, IA, USA) and maintained according to an established method [11,12,13]. HUVEC cells used for our study were between culture passages 3 and 5. MA, LPS (from *Escherichia coli*), dimethyl sulfoxide (DMSO), penicillin G, and streptomycin were obtained from Sigma Chemical Co. (St. Louis, MO, USA). Human HO-1 siRNA duplexes were purchased from Santa Cruz Biotechnology (Santa Cruz, CA, USA).

### 2.2. Animal Care, LPS-Injected Lung Injury Model, and Analysis of Bronchoalveolar Lavage Fluid (BALF)

Male C57BL/6 mice, 6 or 7 weeks old (average weight: 27 g), were obtained from Orient Bio Co. (Seongnam, Korea) and used after an acclimatization period of 12 days, as previously described [14,15]. LPS (15 mg/kg i.p.) with normal saline (the vehicle control) was injected into the peritoneal space using a 28-gauge needle. Six hours after the LPS injection, MA (0.07–0.7 mg/kg) was intravenously administered. This protocol was approved by the Animal Care Committee at Kyungpook National University (IRB No. KNU 2017-102). BALF was collected using an intratracheal phosphate buffered saline (PBS) injection by subsequent gentle aspirations. The BALF was pelleted at 3000 rpm for 10 min at 4 °C, and the supernatant was stored at −80 °C for subsequent studies. 

### 2.3. Enzyme-Linked Immunosorbent Assays (ELISA) for iNOS, PGE2, HO-1, STAT-1 (Both Total and Phosphorylated Forms), TNF-α, and IL-1β

ELISA kits (R&D Systems, Minneapolis, MN, USA) were used to measure the levels of the total and phosphorylated STAT1 protein. The supernatants of the cell culture media were used to analyze the concentrations of PGE2, HO-1, IL-1β, TNF-α, and iNOS (Aviva Systems Biology, San Diego, CA, USA) using ELISA kits. 

The supernatants of the cell culture media were used to analyze the concentrations of PGE2, HO-1, IL-1β α, and TNF-α, using ELISA kits from R&D Systems, whereas iNOS was analyzed using ELISA kit from Aviva Systems Biology, San Diego, CA, USA.

### 2.4. Nitrite Determination 

The level of nitrite (NO_2_^–^) in the medium was measured to estimate the level of nitric oxide. The culture supernatant was mixed with an equal volume of the Griess, and the mixture was incubated for 15 min at room temperature. The reactions were analyzed on a microplate reader (λ = 540 nm). All measurements were conducted in triplicates. 

### 2.5. Subcellular Fractionation and Western Blotting

The cells were detached and harvested via centrifugation. The cytosolic and nuclear extracts were made on ice, according to a previously described protocol [11]. For Western blotting, antibodies against COX2, Nrf2, iNOS, lamin B, and β-actin (Santa Cruz, CA, USA) were used. Lamin B and β-actin were used as the loading controls for the nuclear and cytosolic extracts, respectively. 

### 2.6. Quantitative Real-Time PCR (qPCR)

RNA was purified with TRI Reagent (Invitrogen, waltham, MA, USA) extraction. The purified RNA was reverse-transcribed with a PX2 Thermal Cycler (Thermo Scientific, waltham, MA, USA) using 0.5 mg/µL oligo(dT)-adapter primer (Invitrogen) and M-MLV reverse-transcriptase (Invitrogen) in a 20 µL reaction mixture. The expression levels of COX-2 and iNOS were normalized against that of β-actin. For PCR analysis, the following sets of primers were designed and used: COX-2 forward: 5′-CCC CAT TAG CAG CCA GTT-3′, COX-2 reverse: 5′-CAT TCC CCA CGG TTT TGA-3′; iNOS forward: 5′-GTT CTC AGC CCA ACA ATA CAA GA-3′, iNOS reverse: 5′-GTG GAC GGG TCG ATG TCA C-3′; β-actin forward: 5′-TCGTGCGTGACATCAAAGA-3′; and β-actin reverse: 5′-CAT ACC CAA GAA GGA AGG CT-3′.

### 2.7. Plasmid Transfection

NF-κB luciferase reporter vector, an ARE luciferase reporter vector, HO-1 siRNA, and nonsense control siRNA using SuperFect (Qiagen, Valencia, CA, USA) were used for transfections. After 4 h post-transfection, the transfection medium was replaced with fresh medium.

### 2.8. ARE Luciferase Reporter Assay

Cells were rinsed with cold PBS and lysed with a lysis buffer supplied with a dual luciferase kit (Promega, Madison, WI, USA). The luciferase activities were measured using a TD-20/20 luminometer (Tumer Designs, Sunnyvale, CA, USA). All the transfections were conducted in triplicate. We present our data as the ratio of luciferase activities of Firefly to Renilla.

### 2.9. Histopathological Examination with Hematoxylin and Eosin H&E Staining

The mice (*n* = 5) were injected with LPS. After 6 h, they were administered with MA (12.4 mg/kg, i.v.) and then euthanized and sacrificed. H&E staining was used to analyze the histopathological changes in the lungs [16]. Pulmonary architecture scores were analyzed from grade 1 to grade 4, as previously described [11]. 

### 2.10. Statistical Analysis

The data are shown as the mean values with a standard deviation (SD) of three independent experiments. When a one-way analysis of variance (ANOVA) indicated a significant difference between different groups, post-hoc analysis of the differences between the individual groups was done with Tukey’s tests. A *p*-value of < 0.05 was considered as statistically significant. 

## 3. Results and Discussion

### 3.1. Effect of MA on Levels of iNOS and COX-2 in LPS-Treated HUVECs

**Figure 1:**
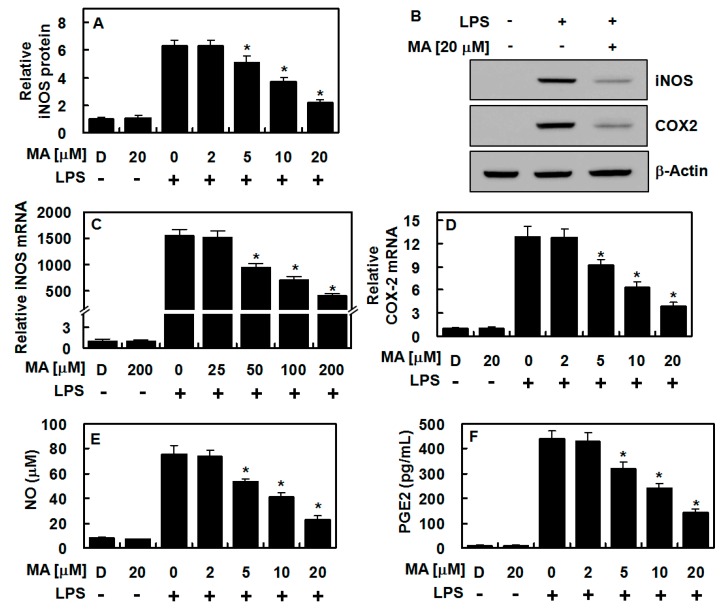
Maslinic acid (MA) suppressed COX-2 and iNOS levels in lipopolysaccharide (LPS)-treated human umbilical vein endothelial cells (HUVECs). After LPS stimulation (1 μg/mL, 6 h), HUVECs were treated with indicated concentrations of MA for 6 h, and the levels of iNOS protein (**A,B**), COX-2 protein (**B**) (Uncropped pictures of the Western blot was shown in Figure S1), iNOS mRNA (**C**), COX-2 mRNA (**D**), NO (**E**), and PGE2 (**F**) were analyzed. The results represent the mean value with SD from three independent experiments conducted in triplicates on different days. D denotes 0.2% DMSO treatment, which was used as the vehicle control. * *p* < 0.05 versus LPS.

To investigate the effect of MA on the expression of genes involved in inflammation, two representative pro-inflammatory proteins, COX-2 and iNOS, were examined. Six hours after stimulation with LPS, HUVECs were treated with different concentrations of MA for another 6 h. The results from qPCR, ELISA, and immunoblot analyses showed that the expression levels of COX-2 and iNOS decreased with administration of MA in a dose-dependent manner (Figure 1A–D). To confirm this, the levels of their corresponding products—PGE2 and NO, respectively—were measured, and they were also found to be reduced following MA treatment (Figure 1E,F). This indicated that MA primarily suppressed LPS-induced NO production by downregulating iNOS expression.

### 3.2. Effect of MA on NF-κB Activity, STAT-1 Phosphorylation, and the Level of HO-1 Protein in LPS-Treated HUVECs

**Figure 2:**
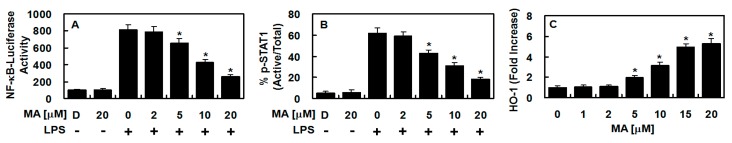
MA suppressed NF-κB activity and phosphorylation level of STAT-1 and upregulated HO-1 protein levels. After LPS stimulation (1 μg/mL, 6 h), HUVECs were treated with indicated concentrations of MA for 6 h. (**A**) NF-κB activity was analyzed in cells that were transfected with NF-κB luciferase reporter vector. (**B**) LPS-mediated phosphorylation of STAT1 (p-STAT1) was measured with ELISA. (**C**) Heme oxygenase (HO)-1 expression from the extracted proteins were analyzed with ELISA. The results represent the mean value with SD from three independent experiments conducted in triplicates on three different days. D denotes 0.2% DMSO treatment, which was used as the vehicle control. * *p* < 0.05 versus LPS.

Because NF-κB is critical for expression of inflammatory genes, we determined whether MA inhibited NF-κB activity. Figure 2A shows that MA inhibited NF-κB luciferase reporter activity in a dose-dependent manner. Since the JAK/STAT signaling pathway plays an essential regulatory role in the expression of COX2 and iNOS in an LPS-activated state [17,18], we investigated whether MA reduced phosphorylation of STAT-1. We indeed found that MA reduced phosphorylation of STAT-1 (Figure 2B). We also found that MA significantly upregulated HO-1 expression (Figure 2C). 

### 3.3. Effect of MA on Nuclear Translocation of Nrf2, ARE Reporter Activity, and Anti-Inflammatory Action

**Figure 3:**
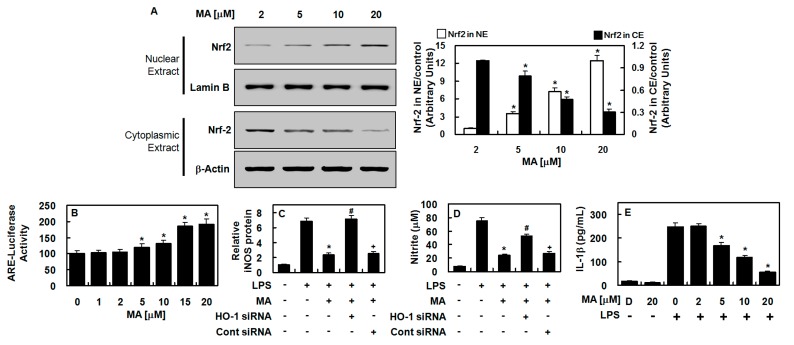
MA induced the nuclear translocation of Nrf2 and anti-inflammatory action in HUVECs. (**A**) HUVECs were harvested, and cytosolic and nuclear fractions were extracted (CE and NE, respectively) after treatment with MA (2–20 μM) for 6 h. Western blotting was employed with indicated antibodies (Left panel), (Uncropped pictures of the Western blot (upper panel and lower panel) was shown in Figures S2 and S3, respectively), and the densitometric intensity of Nrf2 normalized to Lamin B or β-actin is shown (Right panel). (**B**) ARE luciferase reporter activity was measured with lysates from cells transfected with ARE. (**C,D**) HO-1 expression was suppressed with siRNA to determine whether MA-mediated HO-1 expression was responsible for iNOS (**C**) and NO (**D**) inhibition. (**E**) IL-1β concentrations were measured with an ELISA kit. The results represent the mean value with SD from three independent experiments conducted in triplicates on three different days. D denotes 0.2% DMSO treatment, which was used as the vehicle control. * *p* < 0.05 versus LPS, # *p* < 0.05 versus LPS + MA, or + *p* < 0.05 versus LPS + MA + HO-1 siRNA.

Since the expression of antioxidant proteins, including HO-1, is dependent on Nrf2, we investigated whether MA activates Nrf2. We found that MA induced the nuclear translocation of Nrf2 (Figure 3A) and increased ARE luciferase reporter activity (Figure 3B). To confirm that the MA-induced inhibition of iNOS expression was mediated by the upregulation of HO-1, a small interference RNA (siRNA) suppression of HO-1 was conducted. With the suppression of HO-1, the suppression of iNOS expression and NO production by MA were considerably reverted to MA untreated levels (Figure 3C,D). This indicates that MA promoted HO-1 expression, partly through the downregulation of iNOS expression. The anti-inflammatory effect of MA was also validated by its suppression of IL-1β production in LPS-treated HUVECs (Figure 3E). 

### 3.4. Effect of MA on TNF-α and iNOS Protein in an LPS-Induced Lung Injury Model 

**Figure 4:**
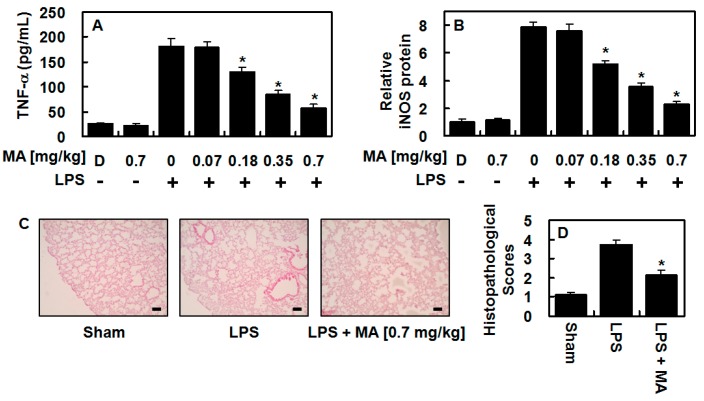
In LPS-injected mice, MA suppressed the levels of TNF-α and iNOS and alleviated lung tissue injury. LPS (15 mg/kg, i.p.) was injected first and MA (0.07–0.7 mg/kg, i.v.) was administered 6 h after LPS injection. Control mice were not injected with LPS. Five mice were used for each LPS and MA treated/LPS nontreated, control group. Lung tissue and bronchoalveolar lavage fluid (BALF) were collected when the mice were sacrificed 1 d after the LPS challenge, and the levels of TNF-α (**A**) and iNOS (**B**) were analyzed. The results represent the mean value with SD from three independent experiments performed in triplicates on three different days. D denotes 0.2% DMSO, which was used as the vehicle control. (**C**) Hematoxylin and eosin (H&E) staining of lung tissues from each group was conducted and representative images from three independent experiments performed on three different days are shown. The bar represents 100 μm. (**D**) Histopathological scores for the lung tissue were recorded as described in the methods section. * *p* < 0.05 versus LPS.

Next, we investigated whether MA produced an anti-inflammatory effect in vivo. As shown in Figure 4A, TNF-α production in LPS-treated BALF was significantly reduced. Based on an estimated circulating blood volume of 72 mL/kg for mice [19,20,21] and an average weight of 27 g for the mice used in this study, the average blood volume was calculated to be 2 mL. Thus, an injection of 0.07, 0.18, 0.35 or 0.7 mg/kg of MA yielded an estimated concentration of up to 2, 5, 10 or 20 μM in the peripheral blood, respectively. In the lung tissue, iNOS protein level was almost completely reversed following the administration of MA (Figure 4B), indicating an anti-inflammatory effect of MA in vivo. Histological analysis also revealed that MA considerably alleviated the pulmonary injury caused by LPS (Figure 4C,D).

## 4. Conclusion

Our study demonstrated that MA induced the expression of HO-1 in HUVECs, in both tim- and dose-dependent manners. Notably, MA also inhibited LPS-induced COX2/PGE2 and iNOS/NO levels and NF-κB activity. NF-κB is involved in numerous processes, such as cell proliferation, cell adhesion, developmental signals, the regulation of differentiation, and protection against cell apoptosis [22]. Moreover, during periods of inflammation, NF-κB regulates immune responses by the production of pro-inflammatory mediators. High levels of NO are also involved in airway inflammatory responses through the regulation of chemokine secretion, while LPS-induced expression of iNOS and COX-2 requires sufficient activation of NF-κB. MA is a natural pentacyclic triterpene, which can be found in various natural sources [5,6]. Studies investigating the mechanism of MA in inflammation showed that it regulates the production of reactive species and the expression of the corresponding inflammatory enzymes [10,23,24]. MA also reduces the cellular cytokines, such as NF-κB, COX2 [25], matrix metalloproteinases (MMPs), urokinase-type plasminogen activator (uPA), and hypoxia inducible factor-1α (HIF-1α), which promote tumor growth and progression [25]. Thus, our data indicate that the MA-induced inhibition of expression of pro-inflammatory mediators (iNOS, NO, COX2, and IL1-β) and the MA-induced production of HO-1 were mediated through the suppression of NF-κB activity. Additionally, the MA-induced inhibition of expressions of TNF-α and iNOS in the BALF of LPS-treated mouse may be mediated by HO-1 induction. Collectively, based on our results and previously published reports, we propose that MA exerts its anti-inflammatory action through the regulation of HO-1 induction. The induction of HO-1 inhibits NF-kB activation and/or oxidative enzyme activity, which reduces the availability of the substrates for COX2 and STAT-1 phosphorylation. Furthermore, MA-induced translocation of Nrf2 from cytosol into the nucleus by an increased Nrf2-ARE binding activity results in the reduced inflammatory cytokines.

Our conclusion is further supported by the fact that specific RNAi-mediated suppression of HO-1 significantly reversed the inhibitory effect of MA on iNOS expression and NO production. Thus, our present study revealed that MA effectively promotes the expression of HO-1 and reduces the pro-inflammatory mediators in LPS-treated HUVECs, and iNOS and TNF-α levels in LPS-induced mice lung tissue. These findings collectively suggest the significance of HO-1 in suppression of the inflammatory processes and highlight TNF-α as a putative downstream molecule of HO-1. Thus, we suggest MA as a potential candidate for use against inflammatory disorders, particularly pulmonary injury.

## Supplementary Materials

The following are available online at https://www.mdpi.com/2076-3921/9/2/106/s1, Figure S1: Uncropped pictures of the Western blot shown in Figure 1B; Figure S2: Uncropped pictures of the Western blot shown in Figure 3A (Upper); Figure S3: Uncropped pictures of the Western blot shown in Figure 3A (Lower).
