# Supplementary material for: Maslinic Acid Ameliorates Inflammation via the Downregulation of NF-κB and STAT-1
Source: Antioxidants (Basel). 2020 Jan 25;9(2):106. doi: 10.3390/antiox9020106 (PMC7070941; doi:10.3390/antiox9020106)
Supplement: Supplementary file 1 [file antioxidants-09-00106-s001.pdf]

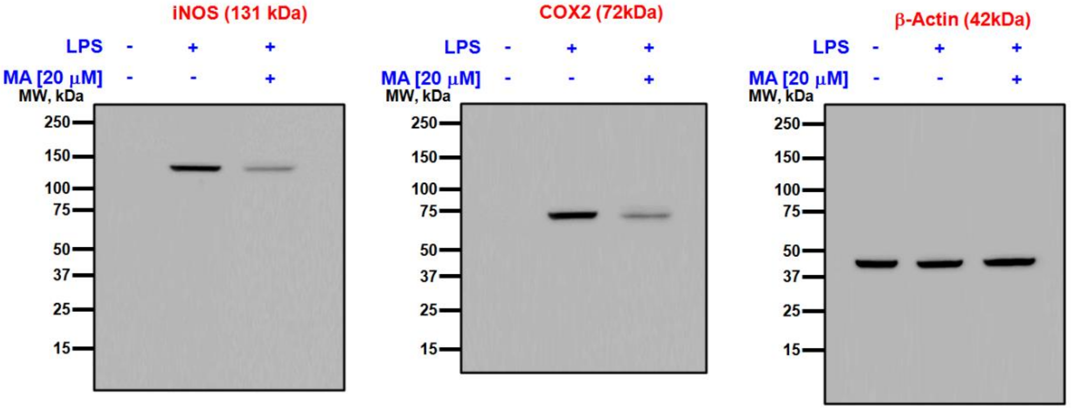

Supplementary Figure 1 (for Fig 1B)

Figure S1: Uncropped pictures of the Western blot shown in Figure 1B.

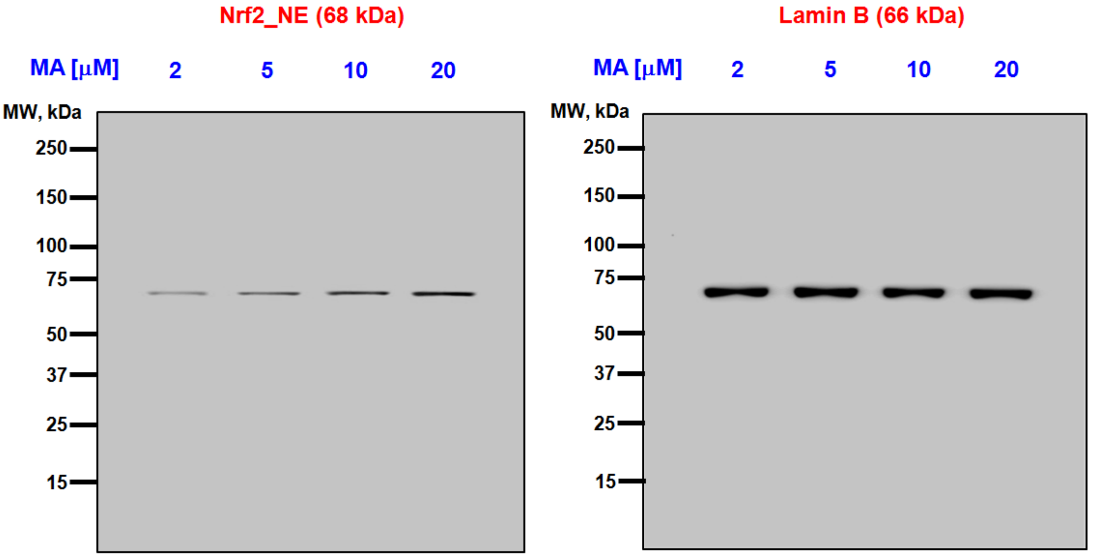

Supplementary Figure 2 (for Fig 3A upper, Nuclear Extract)

Figure S2: Uncropped pictures of the Western blot shown in Figure 3A (Upper)

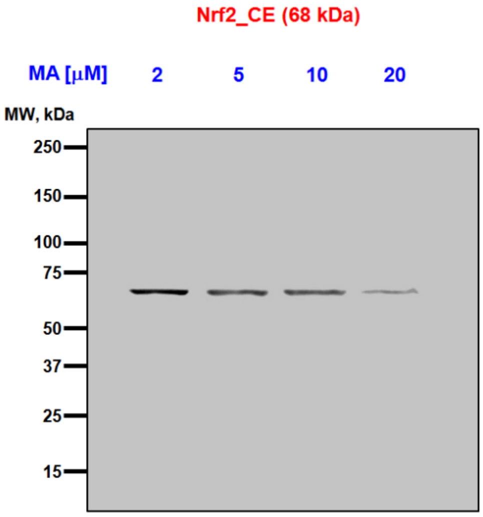

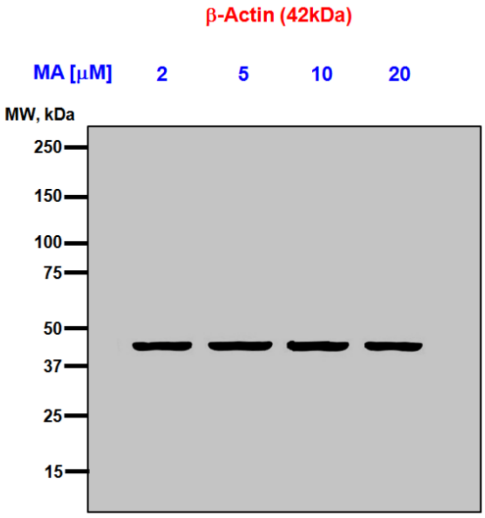

Supplementary Figure 2 (for Fig 3A lower, (Cytoplasmic Extract)

Figure S3: Uncropped pictures of the Western blot shown in Figure 3A (Lower).
